# Surgical outcomes of laparoscopic hysterectomy with concomitant endometriosis without bowel or bladder dissection: a cohort analysis to define a case-mix variable

**DOI:** 10.1186/s10397-018-1039-3

**Published:** 2018-03-16

**Authors:** Evelien M. Sandberg, Sara R. C. Driessen, Evelien A. T. Bak, Nan van Geloven, Judith P. Berger, Mathilde J. G. H. Smeets, Johann P. T. Rhemrev, Frank Willem Jansen

**Affiliations:** 1 Department of Gynecology, Leiden University Medical Centre, Leiden, the Netherlands; 2 Department of Medical Statistics, Leiden University Medical Centre, Leiden, the Netherlands; 3 Department of Gynecology, Haaglanden Medical Centre, the Hague, the Netherlands; 4 Department BioMechanical Engineering, Delft University of Technology, Delft, the Netherlands; 5 Department of Gynecology, Minimally Invasive Surgery, Leiden University Medical Centre, PO Box 9600, 2300 RC Leiden, the Netherlands

**Keywords:** Concomitant endometriosis, Laparoscopic hysterectomy, Case-mix correction, Surgical outcome measures

## Abstract

**Background:**

Pelvic endometriosis is often mentioned as one of the variables influencing surgical outcomes of laparoscopic hysterectomy (LH). However, its additional surgical risks have not been well established. The aim of this study was to analyze to what extent concomitant endometriosis influences surgical outcomes of LH and to determine if it should be considered as case-mix variable.

**Results:**

A total of 2655 LH’s were analyzed, of which 397 (15.0%) with concomitant endometriosis. For blood loss and operative time, no measurable association was found for stages I (*n* = 106) and II (*n* = 103) endometriosis compared to LH without endometriosis. LH with stages III (*n* = 93) and IV (*n* = 95) endometriosis were associated with more intra-operative blood loss (*p* = < .001) and a prolonged operative time (*p* = < .001) compared to LH without endometriosis. No significant association was found between endometriosis (all stages) and complications (*p* = .62).

**Conclusions:**

The findings of our study have provided numeric support for the influence of concomitant endometriosis on surgical outcomes of LH, without bowel or bladder dissection. Only stages III and IV were associated with a longer operative time and more blood loss and should thus be considered as case-mix variables in future quality measurement tools.

## Background

Measuring surgical outcomes to improve quality of health care has received increasing attention over the past decades. Consequently, many national registration systems have been developed to collect hospital data and compare surgical outcomes between hospitals or even surgeons. An important limitation of most of these registrations is the lack of correction of case-mix variables [1]. Case-mix variables are defined as specific patient characteristics that are known to independently influence surgical outcome measures and that are potentially explaining the differences in outcomes between hospitals and/or surgeons [1, 2]. Thus, for an appropriate and reliable interpretation of the surgical outcomes, a case-mix correction is absolutely mandatory.

Specific for the laparoscopic hysterectomy (LH), studies have demonstrated that BMI, uterine weight, and previous procedures influence surgical outcome measures [1, 2]. As a result, a case-mix correction for these variables has been recommended when comparing outcomes of LH [1, 2]. In a recent systematic review on this topic, pelvic endometriosis was also found to be a potential factor influencing surgical outcomes of LH [2]. However, no further conclusions on concomitant endometriosis could be drawn in this review as available evidence was limited. Indeed, only three retrospective studies were found that demonstrated an association between endometriosis and prolonged operative time and increased complication risk of LH [3–5]. Furthermore, none of these studies applied a correction for the known case-mix variables, which may have potentially resulted in an over- or underestimation of the impact of endometriosis.

The objective of this study was firstly to analyze to what extent concomitant endometriosis influences surgical outcomes of LH and secondly, to determine if concomitant endometriosis should be considered as case-mix variables in future quality tools.

## Methods

In this study, all hysterectomies registered, between April 2014 and September 2016, in the web-based application of QUSUM (https://www.qusum.org) were included [1]. Detailed information on this database has been described elsewhere [1], but briefly, data were obtained by asking gynecologists performing laparoscopic surgery to register anonymously their consecutive LH’s for benign indication and low-grade malignancy (e.g., cervical and endometrial dysplasia). All Dutch gynecologists performing LH’s were recruited via a personal e-mail invitation, and gynecologists from abroad were asked to participate in the study at international meetings and conferences. The Institutional Review Board (IRB) of Leiden University Medical Centre, Leiden, the Netherlands reviewed the study and exempted it from IRB approval.

To evaluate the association between endometriosis and surgical outcomes, we retrospectively compared surgical outcomes of patients undergoing LH with and without concomitant endometriosis. Only procedures with hysterectomies as only surgery were considered, including LH’s with concomitant adnexal surgery. Hysterectomies including bowel or bladder dissection of endometriosis were excluded. Indeed, it did not seem fair to compare these operations to ‘simple’ LH’s for benign indication or low-grade malignancy.

We considered as primary outcomes the following three surgical outcome measures: intra-operative blood loss (ml), operative time (listed as time from incision to closure), and complications (up to 6 weeks postoperatively). Complications were defined according to the internationally accepted classification of the Dutch Society of Obstetrics and Gynecology (Appendix 1) [6]. The severity of the complications was graded according to the following classification: level A, recovery without reoperation; level B, reoperation indicated; level C, permanent injury and loss of function; level D, death [6]. Only reactive conversions to laparotomy, as defined by Blikkendaal et al., were included [7]. To adequately compare LH’s with and without endometriosis, a correction of the three known case-mix variables BMI (kg/m^2^), uterine weight (grams), and previous abdominal procedures (previous laparotomy and therapeutic laparoscopy) was applied [2].

For each LH registered in the QUSUM database, we abstracted the data of the three primary outcomes, the three case-mix variables, and the following baseline characteristics: the presence and stage of concomitant endometriosis, the age of the patients at procedure (years), the type of hysterectomy (total laparoscopic hysterectomy (TLH)), supracervical laparoscopic hysterectomy (SLH), laparoscopic-assisted vaginal hysterectomy (LAVH) and robotic hysterectomy (RH)), and the experience of the surgeons (years of experience, number of LH’s per year, and total number of LH’s performed). Gynecologists were asked to enter the information on experience at initial registration in the web application [1].

Endometriosis was classified into four stages, according to the revised definition of the American Society for Reproductive Medicine [8]. Stage I was defined as minimal endometriosis with only superficial lesions and a few filmy adhesions; stage II, the mild variant, included additional deep lesions in the Douglas cavity; stage III was a moderate stage where, in addition to the previous stages, endometriomas on the ovary are observed together with more dense adhesions. Finally, stage IV, the severe endometriosis, included large endometriomas and extensive adhesions.

### Statistics

For the statistical analysis, SPSS version 23.0 (SPSS Inc., Chicago, IL, USA) and R statistical software version 3.3.1 were used [9]. Categorical data were presented as frequency with percentages (%) and continuous data were presented as mean with standard deviation (SD) or as geometric mean with geometric standard deviation (GSD), if data were skewed. To assess significant differences between the baseline characteristics of the group with endometriosis and the one without, independent sample *t* tests, Fisher’s tests, and chi-square with trends were used as appropriate. Statistical significance was defined as a *p* value < .05. A (generalized) linear mixed model regression (univariable and multivariable analysis) was performed to define the association between endometriosis and surgical outcomes. The case-mix variables BMI, uterine weight, and previous procedures were included as co-variables [1, 10]. The characteristic “previous procedures” was dichotomized into no previous procedures or at least one. Data with a skewed distribution were transformed for analysis into log values. We found that blood loss, operative time, and uterine weight needed a log transformation, which resulted in normalization of the distribution. In 55 cases (including five patients with endometriosis) surgeons reported no blood loss after procedure (0 ml). Blood loss was therefore transformed into log(*x* + 1).

To correct for the fact that surgeons entered various procedures, analyses were performed with a random intercept for the performing surgeon. For blood loss and operative time, findings of the regression analyses were presented as percentage increase or decrease with 95% confidence interval (95% CI). The results for complication risk were reported as odds ratio with 95% CI. To make data easier to interpret, an index patient was used to demonstrate the additional effect of the different stages of endometriosis on surgical outcomes. The other case-mix characteristics of this index patient were fixed and based on the mean values of the entire cohort.

## Results

During the study period, a total of 2655 LH’s were analyzed of which 397 cases (15.0%) with concomitant endometriosis. Table 1 gives an overview of the baseline patient characteristics and surgical outcomes. Patients in the endometriosis group were younger (43.5 (7.7) versus 49.5 (11.9), *p* < .001), had lower uterine weight (149.3 (1.8) gram versus 162.5 (2.1), *p* < .001), lower BMI (27.9 (5.5) versus 28.6 (6.4), *p* = .04), and had had more previous abdominal surgeries (61.6 versus 42.9%, *p* < .001).

**Table 1:** Baseline patient characteristics and surgical outcomes of LH with and without endometriosis

	LH with endometriosis *n* = 397	LH without endometriosis *n* = 2258	*p* value
Patient characteristics			
Age, years, mean ± SD	43.5 ± 7.7	49.8 ± 11.9	< .001
BMI, kg/m^2^, mean ± SD	27.9 ± 5.5	28.6 ± 6.4	.04
Uterine weight, gram, geometric mean; GSD*	149.31 ± 1.8	162.5 ± 2.1	< .001
Previous abdominal procedures, *n* (%)			< .001
None	152 (38.3)	1290 (57.1)	
One	124 (31.2)	592 (26.2)	
Two	64 (16.1)	234 (10.4)	
> Two	57 (14.4)	142 (6.3)	
Stage of endometriosis, *n* (%)			
Stage I	106 (26.7)	–	
Stage II	103 (25.9)		
Stage III	93 (23.4)		
Stage IV	95 (23.9)		
Procedure type, *n* (%)			.03
TLH	376 (94.7)	2036 (90.2)	
SLH	13 (3.3)	109 (4.8)	
LAVH	6 (1.5)	88 (3.9)	
Robotic	2 (0.5)	25 (1.1)	
Surgical outcomes			
Blood loss (ml), geometric mean; GSD*	80 ± 3.1	67 ± 3.4	^ɸ^
Operative time (min), geometric mean; GSD*	94 ± 1.5	91 ± 1.5	^ɸ^
Complications, *n* (%)	24 in 22 patients (5.5%)	175 in 153 patients (6.8%)	^ɸ^
Lesion			
Bladder	3 (0.8)	27 (1.2)	
Ureter	1 (0.3)	9 (0.4)	
Vessel	–	1 (< 0.1)	
Bowel	2 (0.5)	7 (0.3)	
Hemorrhage	5 (1.3)	41 (1.8)	
> 1000 mL intra-operative	5 (1.3)	13 (0.6)	
Wound dehiscence	–	6 (0.3)	
Thrombosis	–	3 (0.1)	
Dysfunction ileus	–	2 (0.1)	
Infections	1 (0.3)	50 (2.2)	
Dysfunction incontinence	–	10 (0.4)	
Other	2 (0.5)	11 (0.5)	
Conversion	10 (2.5)	8 (0.4)	
Severity of complications, *n* (%)			.16
Level A–recovery	18 (4.5)	113 (5.0)	
Level B–reoperation	3 (0.8)	39 (1.7)	
Level C–permanent injury	1 (0.3)	0	
Level D–death	0	1 (< 0.1)	

Data are presented as number (percentage) or as mean ± standard deviation or as geometric mean (GM) with geometric standard deviation (GSD)

*Interpretation of the GSD: 95% of the data are expected to lie in the range of GM/(GSD)^2^ to GM*(GSD)^2^

Statistics: independent *t* test for continuous data; Fisher’s test for categorical data; chi-square with trend for number of previous procedures

^ɸ^This means that for every tenfold increase in uterine weight, blood loss and operative time increased by the stated percentages and odds of complication increased by the stated OR

*SD* standard deviation, *GSD* geometric standard deviation, *LH* laparoscopic hysterectomy, *TLH* total laparoscopic hysterectomy, *SLH* supracervical laparoscopic hysterectomy, *LAVH* laparoscopic-assisted vaginal hysterectomy

Most of the hysterectomies performed were TLH (94.7% in the endometriosis group versus 90.2%). The four stages of endometriosis were almost equally divided in the group with endometriosis (stage I: *n* = 106 (26.7%); stage II: *n* = 103 (25.9%); stage III: *n* = 93 (23.4%); stage IV: *n* = 95 (23.9%)). A total of 199 complications occurred in 175 patients (6.6%), including 22 patients with endometriosis. Regarding the severity of the complications, no difference between the group with and without endometriosis was observed (*p* = .16). Specifically, no increased risk was observed in the endometriosis group for the different organ injuries (bladder, ureter, and bowel). No *p* values were calculated for the three primary surgical outcomes, as these data were used in the regression analysis (Table 3).

As demonstrated in Table 2, a total of 93 surgeons registered on average 33 (30.6) procedures during the study period, performed yearly 31 (17.5) LH’s and had on average 6 years (4.5) of experience. As the data were entered anonymously, it is unknown how many hospitals were involved. A significant difference was observed for the overall surgical experience and LH’s performed with and without endometriosis (*p* < .001). In 50.1% of the cases with endometriosis, procedures were performed by surgeons with an experience of at least 200 LH’s, compared to 34.8% in the group without endometriosis. Of note, no significant correlation was observed between the number of LH’s registered in the application and the overall experience of the surgeons (Spearman’s rho = 0.145, *p* = .169, Appendix 2).

**Table 2:** Surgeon’s characteristics

Surgeon characteristics*	Total *n* = 93 surgeons^ɸ^			
Number of procedures registered in QUSUM	33 (30.6), (1–130)			
Number of procedures per year	31 (17.5), (10–140)			
Years of experience	6 (4.5), (0–21)			
Overall experience	160 (158.9), (0–800)			
Total surgical experience for the LH’s with and without endometriosis**	Total *n* = 93 surgeons^ɸ^	LH’s with endometriosis *n* = 397	LH’s without endometriosis *n* = 2258 LH	*p* value
Overall experience				< .001
0–99	42 (45.2)	125 (31.5)	954 (43.4)	
100–199	21 (22.6)	73 (18.4)	518 (21.8)	
200–299	10 (10.8)	99 (24.9)	347 (15.4)	
≥ 300	19 (20.4)	100 (25.2)	437 (19.4)	

*Data are presented as mean (SD), (minimum-maximum)

**Data are presented as number (percentage)

^ɸ^For one surgeon no information on experience was available

Statistics: chi-square test for trend

*LH* laparoscopic hysterectomy

Number of procedures per year is the number of LH they perform an average on a yearly basis

Years of experience is defined as the number of years of experience since they have finished residency

Overall experience is defined as the total number of LH’s performed by a gynecologist during his or her career as attending, including the teaching cases

Table 3 summarizes the association between the different case-mix variables, the stages of endometriosis, and surgical outcomes, using the mixed model regression analysis (univariate and multivariate analysis). For BMI, uterine weight and previous abdominal procedures, significant associations were observed with blood loss and operative time. Regarding endometriosis, we demonstrated that for blood loss and operative time, no measurable association was found for stages I and II endometriosis compared to LH’s without endometriosis (geometric mean blood loss: no endometriosis: 67 (3.4) mL; stage I: 71.4 (3.2), *p* = .48; stage II: 79.0 (2.8), *p* = .10; operative time: no endometriosis 90.6 (1.5) min; stage I: 88.1 (1.5), *p* = .23; stage II: 81.9 (1.5), *p* = .68). Compared to LH’s without endometriosis, LH’s with stage III and IV endometriosis were associated with more intra-operative blood loss (stage III: 70 (3.6), *p* = .01) and stage IV: 106.4 (2.9), *p* = <.001) and a prolonged operative time (stage III: 95.1 (1.5), *p* = <.001 and stage IV: 117.6 (1.6), *p* = <.001). No significant association was found between endometriosis (all stages) and complication rates (*p* = .62).

**Table 3:** Influence of each covariate on blood loss, operative time and complications

		Crude analysis		Adjusted analysis		
Blood loss	(Geometric) mean + (G)SD^§^ or number (%)	Percentage increase (95% CI)	*p* value of univariate analysis	Percentage increase (95% CI)	Index patient* Data expressed in ml (95% CI)	*p* value of multivariate analysis
Patient characteristics		–				
^10^LOG uterine weight^ɸ^	153.6; 2.0			84.8 (74.5–95.4)		< .001
BMI (increase/1 kg/m^2^)	28.5 ± 6.2			2.4 (1.8–3.0)		< .001
Previous procedures		-				
Yes (versus no)	1213 (45.7)			13.1 (4.3–22.6)		< .001
Stage of endometriosis			< .001			< .001
No endometriosis	67.3; 3.4	Reference		Reference	65.7 (57.2–75.6)	
Stage I	71.4; 3.2	4.9 (−16.1–31.2)	.68	7.6 (− 12.3–31.9)	70.7 (55.7–89.7)	.48
Stage II	79.0; 2.8	16.0 (− 7.3–44.9)	.20	18.8 (− 3.2–45.6)	78.0 (61.4–99.2)	.10
Stage III	70.0; 3.6	27.9 (1–62.1)	.04	34.0 (8.0–66.4)	88.1 (68.7–113.0)	.01
Stage IV	106.4; 2.9	98.8 (56.7–152.4)	< .001	114.3 (72.1–166.7)	140.8 (109.4–180.9)	< .001
Operative time	Number (%)	Percentage increase (95% CI)	*P*-value of univariate analysis	Percentage increase (95% CI)	Index patient* Data expressed in min (95% CI)	*p* value of multivariate analysis
Patient characteristics		–				
^10^LOG uterine weight^ɸ^				20.0 (18.1–21.8)		< .001
BMI (increase/1 kg/m^2^)				1.0 (0.8–1.2)		< .001
Previous procedures		–				
Yes (versus no)				3.8 (1.5–6.1)		<.001
Stage of endometriosis			< 0.001			< .001
No endometriosis	90.6; 1.5	Reference		Reference	92.0 (87.4–96.9)	
Stage I	88.1; 1.5	− 4.4 (− 10.1–1.7)	.16	− 3.3 (− 8.6–2.1)	88.9 (82.7–95.7)	.23
Stage II	81.9; 1.5	0.3 (− 5.7–6.7)	.93	1.2 (− 4.3–6.9)	93.1 (86.5–100.3)	.68
Stage III	95.1; 1.5	15.3 (8.0–23.1)	< .001	17.0 (10.3–24.0)	107.7 (99.8–116.2)	< .001
Stage IV	117.6; 1.6	47.6 (38.0–57.6)	< .001	51.0 (42.3–60.3)	139.1 (128.8–150.1)	< .001
Complications	Number (%)	Odds ratio (95% CI)	*p* value of univariate analysis	Odds ratio (95% CI)	–	*p* value of multivariate analysis
Patient characteristics		–				
^10^LOG uterine weight^ɸ^				0.69 (0.56–0.82)		< .001
BMI (increase/1 kg/m^2^)				1.00 (0.98–1.03)		.935
Previous procedures		–				
Yes (versus no)				1.17 (0.87–1.58)		.305
Stage of endometriosis			.72			.62
No endometriosis	153 (6.8)	Reference		Reference		
Stage I (versus no)	5 (2.9)	1.27 (0.55–2.95)	.57	1.27 (0.54–2.98)		.59
Stage II	7 (4.0)	0.69 (0.33–1.43)	.32	0.67 (0.32–1.36)		.26
Stage III	4 (2.3)	1.23 (0.48–3.10)0.76	.67	1.16 (0.45–2.96)		.76
Stage IV	6 (3.4)	(0.34–1.65)	.48	0.67 (0.30–1.50)		.34

^§^Interpretation of the geometric standard deviation (GSD): 95% of the data are expected to lie in the range of GM/(GSD)^2^ to GM*(GSD)^2^

*Index patient with BMI of 28.5, uterine weight of 161.3 g, and 50% previous procedures

^ɸ^This means that for every tenfold increase in uterine weight, blood loss, and operative time increased by the stated percentages and odds of complication increased by the stated OR

Statistics: linear mixed model (for outcome blood loss and operative time) and generalized linear mixed model (with logistic link function) (for outcome complications)

*95% CI* 95% confidence interval

BMI body mass index, *GM* geometric mean, *GSD* geometric standard deviation

For an index patient with stage IV endometriosis, a mean intra-operative blood loss of 140.8 ml (109.4–180.9) was expected compared to a mean of 65.7 ml (57.2–75.6) for a patient with the same characteristics but without endometriosis. Also, an additional mean operative time of 47 min was demonstrated for an index patient with stage IV endometriosis compared to a patient with the same characteristics but without endometriosis (139.1 min (128.8–150.1) versus 92 min (87.4–96.9)).

## Discussion

### Main findings

In this present study, we demonstrated that LH with concomitant stages I or II endometriosis had no measurable and clinical relevant associations with surgical outcome measures compared to LH without endometriosis. Stages III and IV endometriosis, however, appeared to be of influence for the outcomes blood loss and operative time.

### Strengths and limitations

A limitation of our study was that gynecologists registered themselves the procedures and that these data could not be verified as entered anonymously. As a result, we cannot guarantee that the surgeons included all their consecutive cases. Yet, this is similar to daily clinical practice where data are also entered by the surgeons. Moreover, previous studies have shown that these self-reported data are often accurate and reliable [10, 11].

Another limitation is the fact that information regarding the setting, the indication of surgery, or the participation of a fellow or resident during the procedure was missing. This might have biased the outcomes. Although these data would have been interesting, the baseline characteristics (BMI, uterine weight, and previous procedures), known to influence outcomes were analyzed and therefore we believe our comparison is reliable. Regarding the participation of a trainee we believe that it is the responsibility of the principle surgeon to judge if his or her involvement remains acceptable and therefore, no correction for this factor was applied. Additionally, it would have been interesting to know if surgeons knew preoperatively of the presence of endometriosis as this might be relevant for the choice of the surgeon and the surgical planning.

Although the outcome “previous surgeries” has been demonstrated to be associated with worse surgical outcome [2, 10], collecting data on adhesions would have been interesting as well. The advantage of the registration we used was that the number of previous procedures was an objective measure, whereas adhesions and their grading would have been more at risk for intra-observer variations [12]. Finally, in our study, surgery with bowel and bladder dissection of endometriosis was excluded. This might have resulted in an underestimation of the impact of endometriosis on surgery in general. Our data should thus not be generalized to all endometriosis cases but are limited to LH with concomitant endometriosis. Because the aim of our study was to determine the influence of concomitant endometriosis during LH, we believe that it would not be correct to compare these advanced cases to hysterectomies for benign indication or low-grade malignancy. Similar studies should be performed analyzing specifically surgical outcomes of procedures with bladder and/or bowel dissections. Strengths of this study included the relatively large database and number of cases with endometriosis. Also, the high number of surgeons with varying experience adds to the generalizability of the data.

### Interpretation

Although endometriosis is often mentioned as complicating factor during LH, the additional surgical risks associated with LH’s with concomitant endometriosis have not been well established. Yet, for a reliable interpretation and comparison of surgical outcome measures between surgeons and/or hospitals, numeric support of the impact of concomitant endometriosis during LH is necessary. This is also relevant for determining if concomitant endometriosis should be considered a case-mix variable. In this study, we firstly demonstrated that stages III and IV endometriosis were associated with more intra-operative blood loss compared to the group without endometriosis. This finding has, to our knowledge, not been observed in previous studies [3–5, 10]. Although the amount of blood loss was twice as much in the group with stage IV endometriosis compared to the group without, the mean blood loss was still low (141 mL for stage IV). The clinical relevancy for patients is therefore questionable, yet, for the surgeons, it is important to be aware of this potentially increased amount of blood loss. Furthermore, our finding demonstrated that concomitant endometriosis independently influence surgical outcome and therefore should be taken into consideration when applying a case-mix correction.

In agreement with previous studies [4, 13], stages III and IV endometriosis was associated with a prolonged operative time, up to 47 min for stage IV endometriosis. This finding is important to consider for pre-operatively scheduling but also as prolonged operative time has been associated with increased morbidity [14, 15]. One study even demonstrated that every additional hour of surgery during LH increases the risk of postoperative complications by 22% [15].

Regarding complications, previous studies have demonstrated an increased risk for LH’s with endometriosis compared to LH’s without endometriosis. [3–5, 13] Specifically for LH’s with moderate-severe endometriosis (stages III and IV), one of the studies showed an almost fourfold increase in the risk of complications compared to controls [13]. Interestingly, in our study, no significant difference in complication risk was observed (5.5% for the endometriosis group versus 6.8% for the non-endometriosis group). This could be explained by the fact that in previous studies, patients undergoing bowel and bladder resection were included. Other explanations could be related to the overall low number of complications in our study and hence the lack of power to demonstrate a significant difference and/or the high surgical experience for LH with endometriosis [16]. Indeed, LH’s with stages III and IV endometriosis were significantly more often performed by surgeons with more experience, and this might have affected the outcomes. However, we explicitly did not correct for surgical experience in our model as we aimed to demonstrate how patient’s characteristics independently influence surgical outcomes. In daily clinical practice, surgical experience cannot be used either to justify worse surgical outcomes. We want to underline that it is the responsibility of the surgeon to know his individual limitations when counseling a patient. This pre-operative awareness was reflected in our study as most severe endometriosis cases were performed by the more experienced surgeons, and this selection most probably has improved overall surgical outcomes. However, it is important to keep in mind that a high surgical volume does not necessarily directly stand for better surgical outcomes [1]. Although high surgical experience is often associated with positive outcomes, it is not a guarantee. As such, we would recommend surgeons to monitor their individual surgical performances over time rather than to focus on the number of surgeries Table 4 [1].

## Conclusions

For a reliable comparison of surgical outcomes between hospitals and/or surgeons, it is necessary to correct for the patient characteristics that are independently influencing these outcomes. For LH, previous studies have already demonstrated that a case-mix correction for BMI, uterine weight, and previous procedures is required [1, 2]. The findings of our study have provided numeric support for the influence of concomitant endometriosis on the surgical outcomes of LH. We demonstrated that stages III and IV endometriosis were associated with a longer operative time and more blood loss. These specific stages should thus be considered as case-mix variable for these outcomes in future quality measurement tools.

## Figures and Tables

**Fig. 1:**
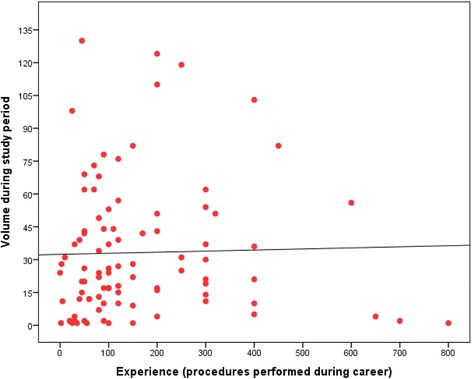
Scatter plot presenting surgeon’s experience versus the number of procedures registered during the study

## Appendix 1

**Table 4:** Complication classification according to the NVOG

Main category	Complication
Infection	- Local - Organ - Systemic
Injury	- Vascular - Bowel - Bladder - Ureter - Other
Wound dehiscence	–
Hemorrhage	- > 1000 mL - Post-operative bleeding
Thrombo-embolism	–
Dysfunction	- Urinary retention - Incontinence - Ileus - Liver - Kidney
Systemic	- Medication error - Adverse drug event - Other
Technical	- Failed procedure - Retained foreign body
Reactive conversion	–
Other	–
